# Comparing a standard and tailored approach to scaling up an evidence-based intervention for antiretroviral therapy for people who inject drugs in Vietnam: study protocol for a cluster randomized hybrid type III trial

**DOI:** 10.1186/s13012-020-01020-z

**Published:** 2020-08-08

**Authors:** Minh X. B. Nguyen, Anh V. Chu, Byron J. Powell, Ha V. Tran, Long H. Nguyen, An T. M. Dao, Manh D. Pham, Son H. Vo, Ngoc H. Bui, David W. Dowdy, Carl A. Latkin, Kathryn E. Lancaster, Brian W. Pence, Teerada Sripaipan, Irving Hoffman, William C. Miller, Vivian F. Go

**Affiliations:** 1 Department of Health Behavior, Gillings School of Global Public Health, 135 Dauer Dr, Chapel Hill, NC 27599 USA; 2 Department of Epidemiology, Institute of Preventive Medicine and Public Health, 1 Ton That Tung St., Dong Da, Hanoi, Vietnam; 3 University of North Carolina Project Vietnam, Lot E2 Duong Dinh Nghe St., Cau Giay, Hanoi, Vietnam; 4 Brown School, Washington University in St. Louis, One Brookings Drive, St. Louis, MO 63130 USA; 5 Vietnam Authority of HIV/AIDS Control, Land 8 That Thuyet St., Ba Dinh, Hanoi, Vietnam; 6 Department of Epidemiology, Johns Hopkins Bloomberg School of Public Health, 615 N Wolfe St, Baltimore, MD 21205 USA; 7 Department of Health, Behavior and Society, Johns Hopkins Bloomberg School of Public Health, 615 N Wolfe St, Baltimore, MD 21205 USA; 8 Department of Epidemiology, College of Public Health, Ohio State University, 250 Cunz Hall, 1841 Neil Ave, Columbus, OH 43210 USA; 9 Department of Epidemiology, Gillings School of Global Public Health, 135 Dauer Dr, Chapel Hill, NC 27599 USA; 10 Division of Infectious Diseases, UNC School of Medicine, 321 S Columbia St, Chapel Hill, NC 27516 USA

**Keywords:** Implementation science, Implementation strategies, Tailoring implementation strategies, Intervention mapping, People who inject drugs, Vietnam, HIV, System navigation, Psychosocial counseling, Intervention, Cost-effectiveness, Economic evaluation

## Abstract

**Background:**

People who inject drugs (PWID) bear a disproportionate burden of HIV infection and experience poor outcomes. A randomized trial demonstrated the efficacy of an integrated System Navigation and Psychosocial Counseling (SNaP) intervention in improving HIV outcomes, including antiretroviral therapy (ART) and medications for opioid use disorder (MOUD) uptake, viral suppression, and mortality. There is limited evidence about how to effectively scale such intervention. This protocol presents a hybrid type III effectiveness-implementation trial comparing two approaches for scaling-up SNaP. We will evaluate the effectiveness of SNaP implementation approaches as well as cost and the characteristics of HIV testing sites achieving successful or unsuccessful implementation of SNaP in Vietnam.

**Methods:**

*Design*: In this cluster randomized controlled trial, two approaches to scaling-up SNaP for PWID in Vietnam will be compared. HIV testing sites (*n* = 42) were randomized 1:1 to the standard approach or the tailored approach. Intervention mapping was used to develop implementation strategies for both arms. The standard arm will receive a uniform package of these strategies, while implementation strategies for the tailored arm will be designed to address site-specific needs.

*Participants*: HIV-positive PWID participants (*n* = 6200) will be recruited for medical record assessment at baseline; of those, 1500 will be enrolled for detailed assessments at baseline, 12, and 24 months. Site directors and staff at each of the 42 HIV testing sites will complete surveys at baseline, 12, and 24 months.

*Outcomes*: Implementation outcomes (fidelity, penetration, acceptability) and effectiveness outcomes (ART, MOUD uptake, viral suppression) will be compared between the arms. To measure incremental costs, we will conduct an empirical costing study of each arm and the actual process of implementation from a societal perspective. Qualitative and quantitative site-level data will be used to explore key characteristics of HIV testing sites that successfully or unsuccessfully implement the intervention for each arm.

**Discussion:**

Scaling up evidence-based interventions poses substantial challenges. The proposed trial contributes to the field of implementation science by applying a systematic approach to designing and tailoring implementation strategies, conducting a rigorous comparison of two promising implementation approaches, and assessing their incremental costs. Our study will provide critical guidance to Ministries of Health worldwide regarding the most effective, cost-efficient approach to SNaP implementation.

**Trial registration:**

NCT03952520 on Clinialtrials.gov. Registered 16 May 2019.

Contributions to the literature

- The proposed study uses rigorous methods, including intervention mapping, to design implementation strategies for an integrated intervention targeting PWID living with HIV in Vietnam. It compares a standard and a tailored approach to scaling up the intervention at the national level.
- This study allows for assessments of the incremental costs of the standard versus tailored approach to implementation. Our cost-effectiveness analyses incorporate prospective, empirical costing of the full implementation process (including all aspects of intervention mapping), not just the intervention itself.
- To inform future scale-up, we will focus on HIV test site-level predictors of successful implementation. These data will provide insight into site characteristics that influence EBI implementation among PWID, thereby informing governments’ decisions about the allocation of limited resources.

## Background

People who inject drugs (PWID) bear a disproportionate burden of HIV infection. Globally, approximately 1.4 million PWID live with HIV, and the prevalence of HIV among PWID has been estimated to be 12.7% [1]. PWID with HIV have high mortality rates compared with their non-drug using counterparts [2]. The mortality difference is largely driven by delayed diagnosis, low uptake, and adherence to antiretroviral therapy (ART) [3]. Most PWID have not been tested for HIV; in some geographic areas, less than 2% of PWID have been tested [4]. In countries with available data, ART coverage among HIV-positive PWID is only 5–67% of PWID living with HIV [1]. PWID lack access to HIV care, medications for opioid use disorder (MOUD), and harm reduction services and limited skills to navigate complex health care systems [4–8]. They also face persistent social barriers, such as stigma and punitive legal systems [5].

Vietnam is a prime country example of an HIV epidemic that has been primarily driven by injection drug use. Vietnam has over 271,000 PWID in the country [9] with an HIV prevalence among PWID in different provinces ranging from 17 to 25% [10–12]. In 2014, the 90-90-90 targets were launched by the Joint United Nations Programme on HIV/AIDS: 90% of all people living with HIV will know their HIV status; 90% of all people with diagnosed HIV infection will receive sustained antiretroviral therapy, and 90% of all people receiving antiretroviral therapy will have viral suppression [13]. Vietnam was the first Asian country to commit to the World Health Organization’s 90-90-90 targets [14], but reaching these targets will require significant efforts to prevent and treat HIV among PWID. Moreover, since funding from international donors for HIV research has dramatically decreased within the last few years [15, 16], it is even more critical for the Vietnamese government to implement effective and cost-efficient HIV interventions.

From 2015 to 2018, we conducted the HIV Prevention Network Trial 074 (HPTN074)—a multi-site study to evaluate the feasibility and efficacy of an integrated systems navigation and psychosocial counseling (SNaP) intervention for HIV-positive PWID on ART use, viral suppression, and MOUD use in Vietnam, Indonesia, and Ukraine [17]. Among PWID with HIV, SNaP led to marked improvements in ART uptake and use, viral suppression, MOUD uptake and use, and mortality [17]. SNaP has potential to curb the epidemic among PWID, but first it needs to be scaled-up to reach a broader population.

Scaling-up evidence-based interventions (EBIs) is often impeded by barriers at different levels, such as constrained budgets, lack of support policies, lack of qualified leaders, and staff as well as cultural resistance to new practice [18, 19]. In reality, EBIs such as SNaP often languish on a shelf or are implemented without careful consideration of barriers in routine care settings [20]. Many countries have recently scaled-up HIV testing and counseling and ART throughout their countries [4, 6] using national level decrees [5, 7]. This top-down, one-size-fits-all decree approach successfully reached many people with HIV [21–23] but has failed to reach PWID [4, 21–23]. The limited success among PWID may be because the national implementation strategy does not address key country-wide barriers or because the implementation approach failed to consider variation by risk groups and clinics. For example, despite the well-documented effectiveness of EBIs such as needle/syringe programs and opioid substitution treatment, the coverage of these EBIs still remained very low among PWID in many parts of the world [4]. This is partly due to policing practices and laws criminalizing drug use and possession implemented by local authorities, which could vary by country, state, or even city [24].

There is a need to identify and evaluate systematic approaches to scaling-up and sustaining EBIs such as SNaP [19, 25, 26], as the best approach to implementing and scaling up such efficacious interventions for PWID in real-world settings is unclear [4, 20]. Scaling-up EBIs will likely require the use of multiple implementation strategies that effectively address multi-level determinants (i.e., barriers and facilitators) of implementation and scale-up [27–29]. Implementation strategies are “methods or techniques used to enhance the adoption, implementation, sustainment, or scale-up of interventions” [28]. The lack of systematic approaches to developing implementation strategies can lead to failed implementation efforts and make them difficult to replicate in other settings [30, 31]. A number of approaches that integrate theory, evidence, and relevant stakeholder perspectives to systematically design and tailor implementation strategies have been identified [32, 33], including intervention mapping (IM) [34–36]. Recent guidance clarifies IM’s role in implementation science by describing how it can be used to adapt interventions and develop implementation strategies [34], and a study underway is examining its potential utility as a method for tailoring implementation strategies at the organizational level [25].

IM applied at the national level may suffice to produce an appropriate national implementation package for SNaP scale up. But implementation determinants at specific HIV testing sites may impede this approach. Thus, it may be useful to apply methods that will help to tailor implementation approaches to address site-specific needs. Tailoring strategies to address contextual needs has face validity [37] and has been shown to be effective at improving health practices [38], but rigorous evaluations that test methods for tailoring strategies and compare tailored and standard multifaceted strategies are needed [28, 32, 38, 39]. Moreover, since the scale up of interventions might be costly and various interventions usually compete for resources and attention from stakeholders [40], evidence of the costs in conjunction with effectiveness of implementation approaches is also required, especially in settings where resources are limited. However, the quantity and quality of economic evaluations of implementation studies remain inadequate [40, 41]. In a systematic review and critical appraisal of health economic methods in implementation research, only 3 (10%) among all studies included were conducted in low- and middle-income countries [40].

To close these gaps, this cluster randomized trial will compare a standard multifaceted approach to implementation to a tailored approach that involves the identification of site-specific determinants combined with implementation facilitation that will help sites identify and apply appropriate implementation strategies. We hypothesize that, compared to the standard approach, the tailored approach will (a) increase site fidelity to SNaP, (b) increase ART uptake among PWID, and (c) be cost-effective. Our specific aims are the following:

1. To compare the standard approach with the tailored approach for scaling-up the integrated intervention, SNaP, for PWID in HIV testing sites in Vietnam.
2. To measure the incremental costs of the standard approach compared with the tailored approach for SNaP implementation in Vietnam.
3. To explore the key characteristics of high- and low-performing HIV testing sites for SNaP implementation in each study arm.

In this protocol paper, we present the conceptual framework, study design, and randomization scheme of the study. We also describe the participants, intervention approaches, outcomes, data collection, and analysis. Finally, we discuss the strengths and weaknesses of the trial, as well as the priorities in the field of implementation science that it addresses. We followed the STARi checklist for implementation intervention [42] and the CONSORT checklist for cluster randomized controlled trial [43] (Additional file 1).

## Methods

### Guiding conceptual frameworks

**Fig. 1:**
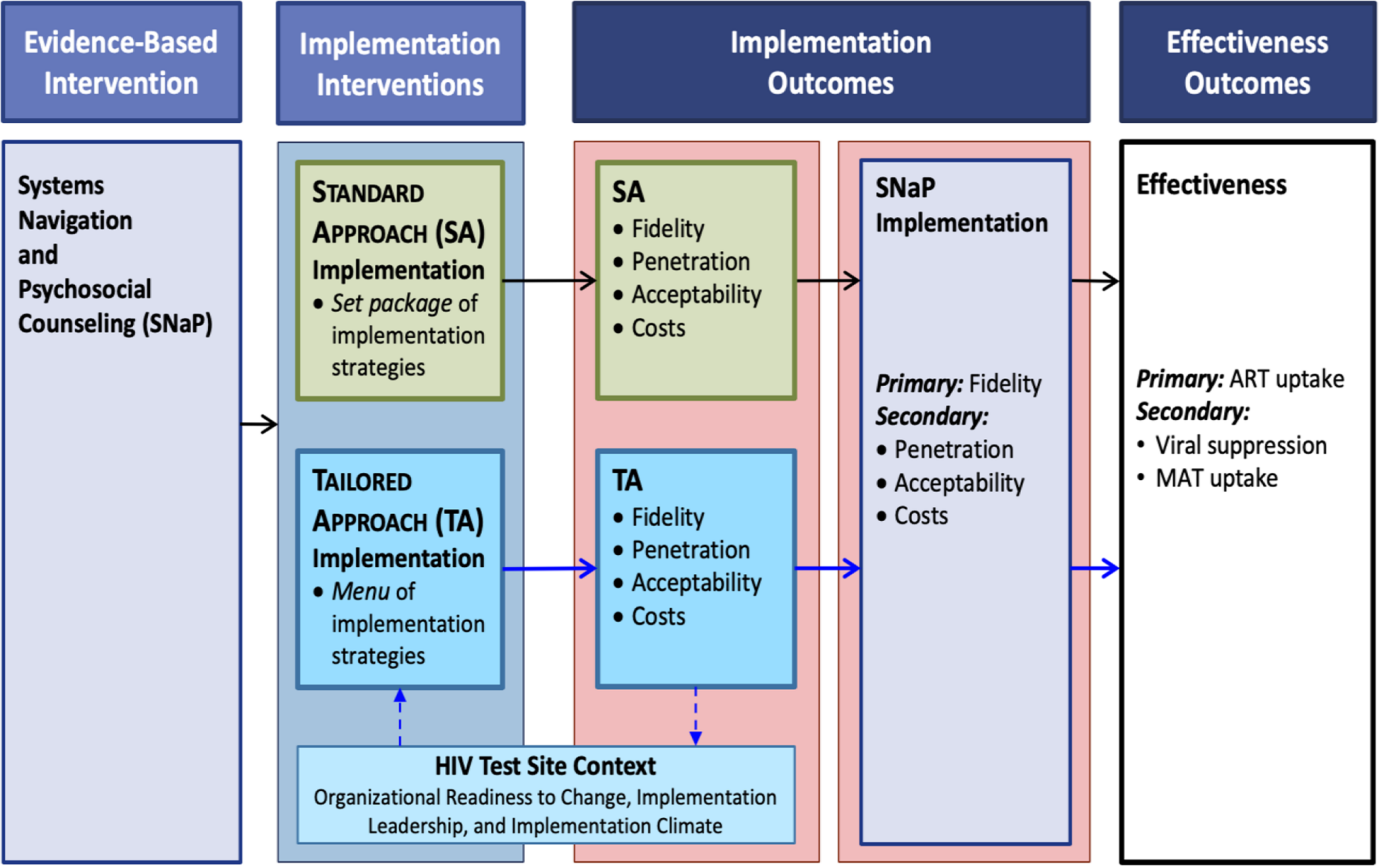
Conceptual framework (adapted from Proctor’s framework [44])

As specified in the conceptual framework (Fig. 1), both standard and tailored implementation approaches are expected to have an effect on SNaP implementation. High acceptability, fidelity, penetration of SNaP implementation will lead to better effectiveness outcomes such as ART uptake, MOUD uptake, and viral suppression. The study is guided by conceptual frameworks to inform implementation processes (intervention apping) [36], identify implementation barriers, and facilitators (Consolidated Framework for Implementation Research; CFIR) [45], and assess implementation outcomes [44, 46]. IM provides a systematic process to yield standard and tailored approaches to implementation. CFIR is a comprehensive framework that identifies 39 different factors in five domains that influence implementation outcomes. In this study, we focus primarily on one of the five CFIR domains, the “inner setting,” defined as the clinic or organizational context in which the intervention will exist [47]. We will measure the inner setting characteristics that influence implementation of SNaP and are likely to vary across sites. These include the age and size of the test site; norms of the test site; organizational readiness to change; implementation leadership, and implementation climate [45, 48–51]. We hypothesize that tailoring implementation strategies to address test site context will improve test site context and lead to better implementation and effectiveness outcomes. The implementation outcomes framework [44] guides our assessment of key implementation outcomes: acceptability, fidelity, penetration, and cost. The effectiveness of SNaP scale-up will be assessed by the implementation processes as operationalized through both arms. As depicted in the conceptual framework (Fig. 1), these implementation outcomes are in relation to both implementation approaches and SNaP itself. First, the two implementation approaches must be acceptable to stakeholders and implemented well (e.g., with fidelity), which in turn, affects SNaP implementation. Second, successful implementation of SNaP, with high fidelity, penetration, and acceptability will lead to better effectiveness outcomes (ART uptake).

### Study design

**Fig. 2:**
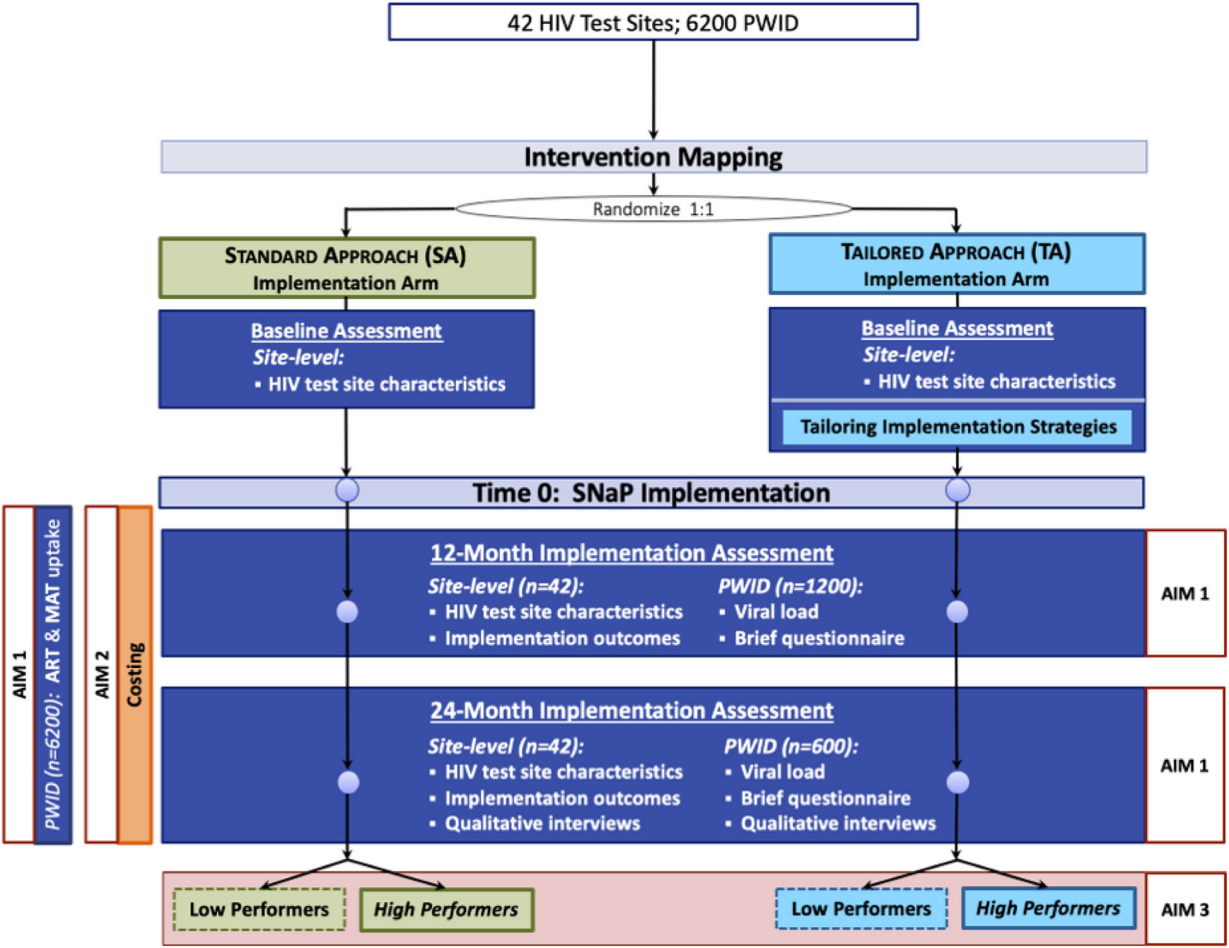
Study design overview and randomization scheme

This is a hybrid type III study that has a dual focus on effectiveness and implementation outcomes [52]. We will conduct a cluster randomized controlled trial in 42 HIV testing sites in 10 Vietnamese provinces with high HIV prevalence among PWID. HIV testing sites are the unit of randomization. The sites will receive either standard or tailored approach with 1:1 allocation to each study arm (Fig. 2).

### Randomization

During the pre-implementation phase, we conducted in-person site visits, where we evaluated the strength of leadership commitment at each site through observation and communication with site leaders. Based on a brief qualitative description of leaders’ engagement and willingness to participate in SNaP, the strength of leadership commitment was classified as either weak or strong. Clinics were then randomly allocated to the two arms at a 1:1 ratio (21 sites per trial arm), stratified by strength of leadership commitment (weak vs. strong). Random allocation was implemented by study statisticians, who used a random number generator to assign each clinic to an arm at a single point in time. Concealment was not used. Randomization results will be masked to data managers, statistical analysts, and staff who collect and/or manage data on the implementation, effectiveness, and costing outcomes. Randomization was conducted before enrolling participants and asking them to provide informed consent.

### Participants

#### HIV testing sites

We obtained a complete list of the 136 HIV testing sites in Vietnam from the Vietnam Authority of AIDS Control (VAAC), including the number of persons tested per month, positivity rates, and proportion of PWID clients. These data were used to identify proposed HIV testing sites for inclusion in this implementation study. Forty-two HIV testing sites in 10 provinces with the highest PWID HIV prevalence were chosen by the study team. The list of HIV testing sites was reviewed and approved by provincial departments of health and the Vietnam Ministry of Health. The ten selected provinces are located across Vietnam and include Dien Bien, Son La, Thai Nguyen, Phu Tho, Ha Noi, Quang Ninh, Nghe An, Khanh Hoa, Ho Chi Minh City, and Long An.

#### PWID participants

Following Vietnam’s national HIV testing algorithm, people with positive rapid test results have their diagnosis confirmed at the nearest MOH-certified confirmatory laboratory, using the standard confirmation HIV test [53]. In this study, eligible PWID participants at chosen HIV testing sites must test positive using the standard confirmatory test; be 18 years or older; report a history of injection drug use in the past 6 months; live in the HIV treatment catchment area and be willing to provide informed consent. The total duration of recruitment is 18 months. Up to 6200 HIV-positive participants will be recruited for medical record assessment at baseline. Of those, 4700 will be asked to provide consent only for medical record assessment and will not be re-contacted. The remaining 1500 participants will be enrolled in a subsample cohort for detailed assessments at baseline, 12, and 24 months, including questionnaires and viral load determination. The sub-sample will be re-contacted for interview and assessment of viral suppression 6–12 months after site implementation. The group enrolled in the first 12 months after site implementation will also have 18–24-month assessments.

#### HIV test site directors and staff

Eligible participants include directors, navigators, counselors, and other staff at HIV testing sites who are willing to provide consent. Six staff at each site will be enrolled and asked to complete brief quantitative surveys at pre-implementation, 12, and 24 months post-implementation. These surveys will be administered online to assess site context measurements, including organizational readiness to change, implementation leadership, and implementation climate. Organizational readiness to change will be measured using the Organizational readiness for implementing change scale—a 12-item scale that evaluates readiness for implementation [54]. Implementation leadership will be measured with the Implementation Leadership Scale, which assesses four dimensions of leadership in EBI implementation: being proactive, knowledgeable, supportive, and perseverant [55]. Implementation climate will be measured using the 18-item Implementation Climate Scale, which evaluates support, recognition, selection, and rewards for innovation use [55] (Additional file 2).

### Interventions

#### Evidence-based intervention: systems navigation and psychosocial counseling (SNaP)

At the individual level, all PWID with confirmed HIV infection at the HIV testing sites will receive the SNaP integrated intervention that has been demonstrated to be effective in HPTN 074 [17]. The intervention was originally designed by the study team to increase engagement in HIV and substance use treatment among PWID [56]. In this study, the SNaP intervention was modified slightly in accordance with the changes in the healthcare system and HIV policies in Vietnam since the HPTN 074 study, such as the merging of district health centers into district hospitals, or payment of HIV care through health insurance instead of international donor funding. Moreover, to increase the feasibility of scaling up, the contents of the original two counseling sessions were combined into one required session only. The intervention has two components: (1) systems navigation to facilitate engagement and retention in HIV care and MOUD (currently methadone in Vietnam), and to negotiate required laboratory tests (e.g. CD4 counts) or transportation; and (2) psychosocial counseling using motivational interviewing, problem solving, skills building, and goal setting to facilitate ART and MOUD initiation and adherence. Systems navigators will initially meet participants twice, in-person or by telephone, within 8 weeks of enrollment. Psychosocial counselors will provide participants with a minimum of one counseling session within 4 weeks of enrollment, with the first session preferably during post-test counseling. To determine the need for additional sessions, the counselor will use a standardized inventory to assess the participants’ need for counseling on risk reduction, drug treatment entry and retention, HIV medical care, ART and MOUD adherence, depression, alcohol dependence, and social support. Participants will be offered the opportunity for additional booster sessions, if needed, at about 1 and 3 months after enrollment.

#### Process for developing implementation strategies

IM can help improve the design of implementation strategies by incorporating theory, evidence, and stakeholder perspectives to ensure that strategy components effectively address key barriers to change [34]. In this study, we used IM to create a menu of standard implementation strategies selected to be applied to all 42 sites (i.e., the standard implementation package).

The study team worked with local stakeholders to conduct IM through the following steps:

1. Step 1: state outcomes (e.g., implementation outcomes such as fidelity, penetration, acceptability, and costs; effectiveness outcomes such as ART uptake, viral suppression, and MOUD uptake) and performance objectives (e.g., who needs to do what to implement and sustain SNaP).
2. Step 2: identify determinants (barriers and facilitators) of implementation through data from HPTN 074’s qualitative data and formative research, including focus group discussions, and informal interviews with local stakeholders and site visits.
3. Step 3: identify appropriate implementation strategies that can potentially help to address identified determinants and achieve performance objectives, using previously published compilations of implementation strategies and a structured brainstorming process.

For tailored approach sites, the menu of strategies will be further tailored to the needs of each clinic.

#### Standard approach

We have developed a multifaceted implementation strategy that includes 15 “discrete” implementation strategies to be applied to the standard arm (Table 1). All strategies will be implemented by the central team, with appropriate collaboration with provincial Centers for Disease Control (CDC) leaders, site leaders, and site staff. Each strategy will be tracked throughout the implementation process by collecting information on the actors, actions, dose, timing of activities, and outcomes of the strategy [57].

**Table 1:** Characteristics of standard implementation strategies (applied to all sites)

No	Strategy	Actor(s)	Actionˠ	Action target	Dose	Timing
01	Organize a meeting with leaders of the Vietnam Authority of HIV/AIDS Control (VAAC) to introduce SNaP	• The central research team*	• Prepare resources and organize a meeting with VAAC to introduce SNaP and discuss collaboration	• VAAC is the governmental organization in charge of HIV/AIDS care in Vietnam and also one of the main collaborators of the study. It is critical that VAAC understands the SNaP intervention and the resources needed for the study at different levels so that they can provide support for the study at the national level	Once	Before SNaP implementation
02	Develop and share a timeline and plan for field activities annually with VAAC and Hanoi Medical University (HMU)—two local collaborators in Vietnam	• The central research team	• Develop and share annual implementation plan with central collaborators and stakeholders at VAAC and HMU	• Stakeholders at VAAC and HMU need to know the timeline and implementation activities of each year as well as their responsibilities, so that they can collaborate well with the central research team to implement SNaP	Annually	Beginning of each year of the study
03	Collect data on site infrastructures and personnel through site initial assessments	• The central research team • Site leaders and staff	• Design a site assessment tool and send them to sites to be completed. • Complete the assessment and return to central team for analysis (*done by site staff*)	• Data on site infrastructures and personnel is collected from sites to inform the feasibility of implementation strategies	Once	Before SNaP implementation
04	Perform initial site visits to introduce SNaP, discuss cooperation mechanism with leaders and staff at provincial and site levels, and identify appropriate personnel for SNaP implementation	• The central research team • Site leaders and staff	• Visit provincial Centers for Disease Control (CDCs) and sites participating in the study to build the initial rapport with members of the board of directors and site staff • Introduce SNaP and receive comments of sites on the implementation of the SNaP • Discuss the plan for collaboration with the sites to implement SNaP (*done by all actors*) • Identify the list of potential staff to participate in launching events, trainings, and SNaP activities • Explore current routines for antiretroviral therapy (ART) and methadone referral at sites, evaluate sites’ resources and willingness to implement SNaP • Identify appropriate staff of sites to participate in SNaP and seek approval from site leaders (*done by all actors*)	• In order to implement SNaP well, site leaders and staff need to understand the nature of the SNaP intervention, design of an implementation study, timeline, budget, human resources needed, and their roles and responsibilities • Since leadership support is critical to successful implementation of SNaP, provincial and site leaders should be involved in the planning of SNaP and feel empowered • Appropriate staff of sites is identified to participate in launching events, training events and to implement SNaP • The central team understands site characteristics needed to implement SNaP in order to design feasible and acceptable implementation strategies	Once during site visits	Before SNaP implementation
05	Identify a focal point person for each province	• The central research team • Provincial CDC leaders, site leaders, and staff	• Visit provincial CDCs and work with them to identify the focal points of contact for SNaP implementation	• In order to ensure effective implementation of SNaP at the sites, it is important to identify focal points of contact at each provincial CDCs. They will help with coordinating the intervention at the provincial level.	Once	Before SNaP implementation
06	Establish a community advisory board consisting of local stakeholders and people who infect drugs (PWID)	• The central research team • Members of the community advisory board	• Invite the focal points of contact at provincial CDCs and representatives of site staff and PWID in the community to join the community advisory board. • Organize semi-annual meetings with the community advisory board	• Provincial leaders and site staff joining the community advisory board are motivated to help coordinate and provide feedback for SNaP implementation • Local stakeholders and the community of PWID have the opportunity to voice their needs and concerns.	Once before enrollment and every 6 after	Before SNaP implementation and every 6 months after implementation
07	Simplify SNaP procedures to prevent duplicate tasks for site staff	• The central research team	• Explore current routines for ART and methadone referral at sites • Simplify SNaP procedures to ensure the procedures are simple to implement and do not repeat existing procedures at sites	• Workload burden related to SNaP is reduced, so that site staff are more motivated to implement SNaP	Once	Before SNaP implementation
08	Revise the SNaP intervention manual, taking into account changes in the local healthcare systems, HIV care policies, and feasibility of scale-up	• The central research team	• Collect information on the structure of the local health systems and the most current policies related to HIV care in Vietnam • Revise the intervention manual accordingly and add new HIV care policies	• The processes of referral and linking to care are consistent with the most current HIV policies and laws in Vietnam • The implementation of SNaP on a large scale is feasible and acceptable given the structure and operation of healthcare systems at the provincial and district levels • Psychosocial counselors and system navigators are prepared to help clients overcome systematic barriers to accessing HIV care	Once	Before SNaP implementation
09	Conduct regional-level launching events (with VAAC, departments of health, provincial CDC leaders, directors of district health centers)	• The central research team • VAAC leaders, provincial CDC leaders, and site leaders	• Provide an overview of SNaP intervention for local leaders • Give speeches to show local leadership support and willingness to implement SNaP (*done by VAAC and local leaders*)	• All stakeholders understand the nature of SNaP intervention, design of an implementation study, timeline, budget, and human resources needed for the study at sites, so that they motivated to collaborate and implement SNaP. • Leadership support and political will also motivate site staff to participate in SNaP and implement SNaP well	Once for each of the four regions	At the beginning of SNaP implementation
10	Conduct regional trainings	• The central research team • System navigators and psychosocial counselors at sites	• Conduct a 4-day training on the SNaP intervention, knowledge and skills for SNaP delivery, the data collection system and other research procedures	• Site staff understands SNaP intervention and their responsibilities. They are also trained to have the necessary skills and knowledge to implement SNaP well at their sites	Once for each of the four regions	At the beginning of SNaP implementation
11	Monthly payment to provincial CDC staff and site staff	• The central research team • Focal points of provincial CDC • System navigators and psychosocial counselors at sites	• Provide monthly payment to provincial CDC for their coordination of SNaP activities • Provide payment for site staff for administrative costs and successful referral of participants to ART treatment	• The study team provides incentives for provincial CDCs to coordinate SNaP activities at the site level. • Payment for administrative costs and successful referral to ART initiation for participants are also provided so that site staff are motivated to implement SNaP as intended.	Monthly	After enrollment begins
12	Send semi-annual progress reports of SNaP implementation to provincial CDCs	• The central research team • Provincial CDC leaders	• Analyze SNaP progress data and write up reports • Share the progress reports of sites with corresponding provincial CDCs	• Provincial leaders are regularly updated on the progress and results of SNaP. They can take charge of coordinating SNaP activities and providing continuous leadership support.	Semi-annually	Every 6 months after enrollment
13	Integrate plans to address turnover, such as replacing and re-training for new staff	• The central research team • Site leaders and staff	• Inform the central research team of staff turnover and replacement (*done by site leaders and staff*) • Conduct training for new staff	• The central research team needs to be properly informed of turnover events, so that they can provide training for new staff. • It is important that new staff understands their responsibilities and has the knowledge and skills to deliver SNaP as intended.	On-going	After turnover events
14	Organize online booster training session	• The central research team • Site leaders and staff	• Create booster training content based on process evaluation of sites’ implementation of SNaP • Create an online platform and upload training materials • Provide access and guidance to site staff to learn through the online training platform • Participate in online booster training (*done by site staff*)	• The skills of system navigators and counselors are improved and their weaknesses in implementing SNaP are addressed	Two times throughout the study	6 and 14 months after implementation of the first regional training
15	Conduct group calls every 6 months by region or province to share experience and feedback	• The central research team • Site leaders and staff	• Facilitate group calls every 6 months for sites in the same arm	• Site staff knows how SNaP is implemented at other sites and have the platform to share their knowledge and experience	Semi-annually	Every 6 months after enrollment

*The central research team refers to the research team from the UNC Vietnam project in Hanoi, including the project management, leads of tailored approach and standard approach arm, project assistant, data assistant, and evaluation and management officer

ˠActions are implemented by the central research team, unless otherwise specified

*Abbreviations*: *ART* antiretroviral therapy, *CDC* Center for Disease Control, *HMU* Hanoi Medical University, *PWID* people who inject drugs, *SNaP* system navigation and psychosocial counseling intervention, *VAAC* Vietnam Authority of HIV/AIDS Control

#### Tailored approach

HIV testing sites in the tailored condition have access to the same implementation strategies developed for the standard arm. In addition, tailored approach sites will have access to a broader menu of additional strategies identified through the intervention mapping process. In order to explore site-specific targets for change that could be addressed through tailored implementation strategies, we will conduct an assessment of each of the 21 sites in the tailored approach arm, using a structured online survey prior to implementation. Consistent with our guiding determinant framework (CFIR), we will assess inner setting factors that may influence implementation processes, including organizational readiness to change [54], implementation leadership [58], implementation climate [59], and available resources. We have developed a pre-determined list of additional strategies (Table 2) selected from the Expert Recommendations for Implementing Change (ERIC) menu—a relatively comprehensive list of implementation strategies suggested by stakeholders with expertise in implementation research, applied implementation, and clinical practice [60].

**Table 2:** List of optional tailored implementation strategies

Strategy	Description†
Audit and feedback	Collect and summarize SNaP implementation data over a specified time period and give it to leaders within sites to monitor, evaluate, and modify SNaP activities
Conduct cyclical small tests of change	Implement changes in SnaP implementation in a cyclical fashion using small tests of change before taking changes system-wide. Tests of change benefit from systematic measurement, and results of the tests of change are studied for insights on how to do better. This process continues serially over time, and refinement is added with each cycle
Conduct local needs assessment	Collect and analyze data related to the local need for the innovation
Obtain and use participants and family feedback	Develop strategies to increase participants and family feedback on the implementation effort
Organize implementation team meetings	Develop and support teams of staff who are implementing SNaP and give them protected time to reflect on the implementation effort, share lessons learned, and support one another’s learning
Capture and share local knowledge	Capture local knowledge from implementation sites on how site staff made something work in their setting and then share it with other sites
Shadow other experts	Provide ways for site staff to directly observe experienced system navigators and psychosocial counselors deliver SNaP
Facilitate relay of data to implementers	Provide as close to real-time data as possible about key measures of process/outcomes using integrated modes/channels of communication in a way that promotes use of SNaP
Remind implementers	Develop reminder systems designed to help site staff to recall information and/or prompt them to use the SNaP intervention
Non-monetary incentives	Non-monetary strategies to motivate site staff to implement SNaP (recognition, certificate, rewards, etc.)

†Adapted from the ERIC menu [60]

Through a hands-on 2-day intensive training session, we will work with these sites to tailor their implementation strategies to address their site-specific determinants of SNaP implementation. Tailored approach sites will decide which strategies to implement based on their own needs and capacity. In addition, provincial health officials and HIV testing sites in the tailored arm will receive resources and coaching to assist them in tailoring their implementation strategy to address key determinants of change throughout the implementation process. This additional coaching will be conducted through external phone assistance and regular calls with each tailored approach site to discuss site-specific challenges and assess ongoing implementation. If adjustment is needed, site-specific standard operating procedures will be updated by the site and reviewed centrally. These activities are presented as three mandatory additional strategies applied to tailored approach sites (strategies 16, 17, and 18 in Table 3).

**Table 3:** Characteristics of tailored implementation strategies applied to all TA sites

No.	Strategy	Actor(s)	Actionˠ	Action target	Dose	Timing
16	Provide external technical assistance through the phone to tailored approach sites	• The central research team*	• Provide external technical assistance through the phone to tailored approach sites whenever they need help with implementing SNaP • Document all requests for help and support for tailored sites	• Tailored approach sites receive the continuous support they need to implement SNaP well. • All requests for help and the support provided by the central team are properly documented throughout the study	On-going	As needed
17	Conduct 2-day, site-specific training to discuss site-specific barriers and develop implementation plan	• The central research team • Site staff	• Discuss with each site to prioritize their specific barriers, facilitators, and finalize the menu of potential tailored strategies as well as implementation plan • Provide training on how to apply these implementation strategies	• Site-specific barriers and facilitators to SNaP implementation are identified and prioritized based on their importance • Acceptable and feasible implementation strategies to overcome the barriers are developed • Sites have a clear and specific plan to implement these strategies once enrollment starts	Once for each tailored approach site	Before SNaP implementation, after regional training
18	Conduct monthly calls to tailored approach sites to provide continuing support in implementing SNaP	• The central research team • Site staff	• Monitor and track the implementation of strategies • Identify new barriers, facilitators and work with sites to create new implementation strategies if needed	• Progress of the implementation of SNaP and implementation strategies at sites is updated regularly to the central research team • New barriers and appropriate implementation strategies to overcome these barriers at sites are identified to improve the implementation of SNaP • The central research team provides continuous support and builds capacities for sites to implement SNaP and other interventions in the future	Once a month	Every month after enrollment

*The central research team refers to the research team from UNC Vietnam project, including the project management, leads of tailored approach and standard approach arm, project assistant, data assistant and evaluation and management officer

ˠActions are implemented by the central research team, unless otherwise specified

### Outcomes

#### Implementation outcomes

The primary implementation outcome is fidelity—the extent to which SNaP is delivered as intended. Fidelity will be measured at the test site level by assessing each site’s success in completing the three protocol-specified sessions (two navigation, one counseling) within the required 8- or 4-week period, respectively, weighted by the central implementation team’s quality rating of those sessions. Session completion will be assessed by reviewing the navigator and counselor logs, while session quality will be assessed by the central implementation team reviewing and scoring of a random 10% of all forms (navigation) and audio-recordings (psychosocial counseling). Central implementation teams will rate them on a 100-point quality scale from poor to excellent. Reviewers will be masked to the arm when reviewing navigation forms or audio-recordings.

$$ \mathrm{Fidelity}=\%\kern0.5em \mathrm{navigation}\kern0.5em \mathrm{sessions}\kern0.5em \mathrm{completed}\ast \mathrm{average}\kern0.5em \mathrm{quality}\kern0.5em \mathrm{score}+\%\kern0.5em \mathrm{counseling}\kern0.5em \mathrm{sessions}\kern0.5em \mathrm{completed}\ast \mathrm{average}\kern0.5em \mathrm{quality}\kern0.5em \mathrm{score} $$

Secondary implementation outcomes are penetration, acceptability, and implementation costs (Table 4).

**Table 4:** Study outcomes

Outcome	Definition	Measure or scale*
Primary implementation outcome		
Fidelity to SNaP protocol	Delivering SNaP as intended	% system navigation sessions completed × average quality score + % counseling sessions completed × average quality score
Secondary implementation outcomes		
Penetration	% of PWID participants contacted, and enrolled in SNaP	Proportion of newly diagnosed or previously diagnosed and not currently on ART participants at HIV testing sites who are contacted by a navigator and/or counselor and participate in a SNaP session.
Acceptability	Perception that SNaP is agreeable, palatable, or satisfactory.	For PWID participants: acceptability of Intervention Measure scale [61] For site staff: Ottawa Acceptability of Decision Rules Instrument [62]
Implementation costs	Costs of SNaP and its implementation	Directly measured non-research costs, including all costs of implementation
Primary effectiveness outcome		
Uptake of ART	% participants who initiated ART	ART clinic records of consenting participants
Secondary effectiveness outcomes		
Viral suppression	% participants virally suppressed	Undetectable viral load on dried blood spots in the subsample cohort
Uptake of MOUD	% of participants on MOUD	MOUD clinic records of consenting participants

*All measures were adapted to SNaP (Additional file 2)

*Abbreviations*: *ART* antiretroviral therapy, *MOUD* medications for opioid use disorder, *PWID* people who inject drugs, *SNaP* system navigation and psychosocial counseling intervention

#### Effectiveness outcomes

The primary effectiveness outcome is ART uptake among all enrolled PWID. ART uptake is defined as the proportion of participants receiving SNaP with evidence of an ART-related visit in an ART clinic. The secondary effectiveness outcomes include viral suppression and MOUD uptake, assessed among the subsample cohort only (Table 4).

#### Cost-effectiveness measurements

We will conduct an empirical costing study of SNaP—including both approaches and the actual process of implementation itself—from a societal perspective. We will estimate implementation costs prospectively by embedding a trained costing specialist within each IM team, documenting all resources used (e.g., staff training level and time, travel costs, supplies, etc.) and estimating the unit cost of each resource. To estimate the unit health system cost of each intervention, we will perform detailed budgetary analysis including interviews of key staff, review of logbooks/timesheets, and time-and-motion studies to record navigator, counselor, and other staff time. To estimate patient costs, we will administer a survey to a smaller subset of the 1500-participant cohort at baseline, 12 months, and 24 months to collect data on direct and indirect (lost wages, child care, etc.) costs of clinic visits and other elements (e.g., medication side effects) associated with ART, MOUD, and SNaP itself.

#### High- and low-performing sites

In HPTN 074, the Vietnam site achieved 86–88% completion of navigation and counseling sessions in the protocol-specified window, and in our counseling studies, we consistently achieve 90–95% quality scores on supervisor-rated counseling sessions [17]. Using the formula to calculate fidelity, a site completing 90% of navigation sessions with an 80% average quality score and 80% of counseling sessions with an 80% average quality score would receive a total score of 136. Our a priori definition of successful implementation at 24 months is a site with a fidelity score of 136 out of 200 and 70% ART uptake among its newly diagnosed or previously diagnosed and not currently on ART participants.

### Data collection and analysis

#### Formative data collection and analysis (year 1, completed)

We are in year 2 of the study and will start enrollment in 2020. In study year 1, as part of the first step of IM, we conducted 2 rounds of focus group discussions among local stakeholders in Hanoi and Thai Nguyen. The first round explored potential structural barriers to and facilitators of SNaP implementation, while the second round discussed appropriateness and feasibility of proposed implementation strategies. During the pre-implementation phase, we also conducted site initial assessments and site in-person visits. A simple initial survey was sent to sites to explore services provided, site’s internet capacity and office space, staffing structure, current referral protocol, leadership strength, and other relevant characteristics. We also visit all sites to communicate with them in-person about the study and qualitatively evaluate the strength of leadership commitment at each site. After each visit, a brief qualitative description on leaders’ engagement and willingness to participate in SNaP was created by the research team. The information and data collected were used for randomization and to inform feasibility of implementation strategies. We also developed a standardized form for collection of cost and resource-use data and have prospectively collected costs during the first year of the study, delineating these costs as research-related versus necessary for programmatic implementation, on a weekly basis.

#### Data collection and analysis after intervention implementation (year 2–4)

##### Quantitative data

To compare two implementation approaches in Aim 1, interview data will be collected for both site staff and PWID participants (in the subsample cohort) at baseline, 12, and 24 months follow-up visits. Viral suppression data will also be collected for PWID participants in the subsample cohort. For ART and MOUD uptake data, each patient will have a second masked identification number, which is used to link with their records at HIV and methadone clinics. Only masked study identification numbers of participants will be used to extract information from ART and MOUD clinics to confirm ART initiation and MOUD uptake.

For the cost-effectiveness analysis in Aim 2, we will administer quantitative costing questionnaires for PWID participants randomly selected at each of 12 purposively selected sites (six per arm, selected for broad representation of geography, number of clients, and proportion of clients who are PWID) at baseline and 12-month follow-up visit. In addition, we have developed a quantitative costing tracker to document costing for study implementation at the above-site (central) level and questionnaires for assessment of implementation costs at all 42 sites.

For Aim 3, site characteristics will be assessed with exploratory analyses to examine associations with high- and low-performing clinics. We will use a generalized linear model with a logit link and binomial error distribution to assess the dichotomous outcome of high or low performance.

##### Qualitative data

As part of Aim 3, we will conduct six semi-structured in-depth interviews with PWID participants in 12 HIV testing sites (for both low- and high-performing sites in two arms at the 24-month follow-up visits). We will ask participants about barriers and facilitators to uptake of ART, ART adherence, MOUD uptake, attitudes, and experiences in the HIV test site and with navigators and counselors. In these same HIV testing sites, we will conduct semi-structured in-depth interviews with navigators/counselors, site staff, site directors, and HIV providers to understand their exposure to and perceptions of SNaP and to inform the context and processes that may underlie SNaP success and failures. All qualitative interviews will be audiotaped, transcribed, translated, coded, and computerized for analysis. Textual data analysis will involve reading for content, deductive and inductive coding, data display to identify emerging themes, data reduction, and interpretation. Responses of navigators and counselors, test site staff, and test site directors will be compared within and across the staff groups and high versus low performing sites. Finally, both qualitative and quantitative data will be combined and triangulated to understand SNaP and two approaches within high- and low-performing sites.

### Sample size

#### Implementation outcomes sample size calculations

For the primary outcome of fidelity, using two-tailed tests with α = 0.05 and assuming a conservatively large standard deviation of 40, 42 sites will give us 80% power to detect a difference between a fidelity score of 136 (high-performing site) in the tailored arm and 100.5 in the standard arm (corresponds to 75% session completion and 67% average quality ratings).

#### Effectiveness outcomes sample size calculations

We estimate that the intra-cluster correlation coefficient (ICC) may range from 0.01 to 0.05, implying a design effect between 2.5 and 8.4 [63–66]. Using two-tailed tests and α = 0.05, a sample size of 6200 (available sample after accounting for 12% mortality) will have > 80% power to detect a difference as small as 10 percentage points (e.g., 70% vs. 60% ART uptake) if the ICC is as large as 0.05, and as small as 6 percentage points (e.g., 70% vs. 64% ART uptake) if the ICC is as small as 0.01. For viral load, which is based on a sample of *n* = 1200 (available sample after accounting for 20% lost to follow-up), assuming the same ICC of 0.01–0.05, we will have 80% power to detect differences of 10–14 percentage points in viral suppression between arms.

## Discussion

The HIV burden among PWID in low-resource settings will only be reduced when EBIs are effectively implemented at scale. To our knowledge, the proposed study will be the first to use IM to design a multifaceted implementation package for PWID at a national level. It will evaluate the best approach to identifying implementation strategies to scale up the SNaP intervention in a low-resource setting.

The lack of a systematic approach to developing implementation strategies can lead to failure to address determinants of implementation [67, 68]. More systematic and rigorous methods are recommended for the design and tailoring implementation strategies for EBIs, such as concept mapping, group model building, conjoint analysis, and IM [32]. In this study, to develop the standard implementation package, we used IM—a mixed-method framework that provides many advantages. Within the literature on tailoring implementation strategies, the approaches used to identify barriers to implementation vary widely [39]. We applied a mix of quantitative and qualitative methods, including focus group discussions and informal interviews with local stakeholders, quantitative assessments of sites’ characteristics and implementation climate, and in-person site visits to explore potential barriers and facilitators to intervention implementation. In addition to IM, our tailored implementation approach allows for flexibility in developing strategies through a two-step local process: rapid assessment of implementation barriers and facilitators in each site; and selection of site-specific strategies using a pre-identified menu of potential strategies to address barriers. Moreover, our continuous interactions with tailored approach sites through monthly calls will provide additional opportunities to communicate with sites about their specific needs and improve SNaP implementation.

This study addresses priorities in the field of implementation science in multiple ways:

- It is a true cluster randomized effectiveness-implementation trial: most HIV studies are individually randomized and/or measure effectiveness rather than implementation outcomes [69]. Our study will randomize at the site-level and will collect primary and secondary implementation outcomes at the site-level.
- It scales up a highly effective HIV intervention for PWID: few HIV trials for PWID have yielded such promising findings across multiple self-reported and biological outcomes as HPTN 074 [17]. Bringing a successful EBIs to scale with careful implementation assessment has the potential to curb the global PWID HIV epidemic.
- It assesses the role of HIV test site context for implementation: while IM has long emphasized the importance of implementation [25, 34, 35], using IM as a method to tailor implementation strategies has rarely been evaluated. The tailored approach allows site-level variables, such as readiness to change and implementation climate, to be addressed as implementation strategies are selected.
- It incorporates cost-effectiveness to inform policy makers in low-resource settings: even though economic evaluations help guide decision making regarding the allocation of resources to the implementation and scale-up of EBIs, they are not routinely done in implementation studies [41, 70]. This study allows for assessments of the incremental costs of the standard versus tailored approach to implementation. Moreover, our cost-effectiveness aim incorporates prospective, empirical costing of the full implementation process (including all aspects of IM), not just the intervention itself.
- It identifies site characteristics to inform future scale-up: the focus on test site-level predictors of successful implementation will be critical for scale-up efforts of EBIs. These data will provide insight into characteristics that influence successful EBI implementation among PWID, thereby informing governments’ decisions about allocation of limited resources.
- It improves tracking and reporting of implementation strategies: poor reporting of implementation strategies makes it impossible to replicate effective strategies or learn from ineffective strategies [25, 28, 57]. To address these limitations, we will carefully track the agencies’ use of implementation strategies using established methods [71, 72]. We will also report the use of strategies using established guidelines [57], which will involve naming, defining, and specifying implementation strategies in sufficient detail to enable replication in other settings.

The biggest challenge to this study is the logistics of implementing a large, cluster randomized study in 10 different provinces across the country. This challenge has been alleviated by the collaboration of our well-trained and experienced permanent UNC-Vietnam study team and our implementing partners in HMU and VAAC, who will oversee implementation and provide technical assistance. A second concern is our ability to track PWID participants as they initiate ART and MOUD, due to the lack of adequate clinical records in Vietnam. We will reduce this concern by establishing effective communication between HIV testing sites and ART clinics, as well as using masked identification numbers to verify and match participants when reviewing clinical records.

## Conclusions

In summary, PWID need impactful interventions to reduce their HIV-associated morbidity and mortality and slow the broader HIV epidemic. This implementation trial will provide critical guidance to policy-makers worldwide who are responsible for reducing the burden of HIV infection among PWID. Regardless of the outcome, the trial will contribute to the field of implementation science through its examination of implementation and effectiveness outcomes, cost, and characterization HIV testing sites that successfully or unsuccessfully implement the intervention.

## Supplementary information

**Additional file 1.** SNaP checklists of study protocol.

**Additional file 2.** Measurement scales.

## Data Availability

Data collection for this study is ongoing, so no data and materials are currently available.

## Abbreviations

ART: Antiretroviral therapy
CDC: Center for Disease Control
CFIR: Consolidated Framework for Implementation Research
EBI: Evidence-based intervention
HMU: Hanoi Medical University
HPTN: HIV Prevention Trial Network
IM: Intervention mapping
MOUD: Medications for opioid use disorder
PWID: People who inject drugs
SNaP: Systems navigation and psychosocial counseling
UNC: University of North Carolina, Chapel Hill
VAAC: Vietnam Authority of HIV/AIDS Control
