# Supplementary material for: Comparing a standard and tailored approach to scaling up an evidence-based intervention for antiretroviral therapy for people who inject drugs in Vietnam: study protocol for a cluster randomized hybrid type III trial
Source: Implement Sci. 2020 Aug 8;15:64. doi: 10.1186/s13012-020-01020-z (PMC7414564; doi:10.1186/s13012-020-01020-z)
Supplement: Supplementary file 1 — Additional file 1. SNaP checklists of study protocol. [file 13012_2020_1020_MOESM1_ESM.pdf]

RESEARCH
Department of Health and Human Services
National Institutes of Health

NATIONAL INSTITUTE ON DRUG ABUSE

Federal Award Date: 08/23/2018

Grant Number: 1R01DA047876-01
FAIN: R01DA047876

Principal Investigator(s):
VIVIAN F. GO (contact), PHD
WILLIAM C MILLER, MD

Project Title: Scaling up HPTN 074: a Cluster Randomized Implementation Trial of an Evidence-based Intervention for Antiretroviral Therapy for PWID in Vietnam

Chipps, Kati
Grants and Contracts Specialist
104 Airport Drive
Chapel Hill, NC 275991350

Award e-mailed to: resadminosr@unc.edu

Period Of Performance:
Budget Period: 09/01/2018 – 06/30/2019
Project Period: 09/01/2018 – 06/30/2023

Dear Business Official:

The National Institutes of Health hereby awards a grant in the amount of $1,017,449 (see "Award Calculation" in Section I and "Terms and Conditions" in Section III) to UNIV OF NORTH CAROLINA CHAPEL HILL in support of the above referenced project. This award is pursuant to the authority of 42 USC 241 42 CFR 52 and is subject to the requirements of this statute and regulation and of other referenced, incorporated or attached terms and conditions.

Acceptance of this award including the "Terms and Conditions" is acknowledged by the grantee when funds are drawn down or otherwise obtained from the grant payment system.

Each publication, press release, or other document about research supported by an NIH award must include an acknowledgment of NIH award support and a disclaimer such as "Research reported in this publication was supported by the National Institute On Drug Abuse of the National Institutes of Health under Award Number R01DA047876. The content is solely the responsibility of the authors and does not necessarily represent the official views of the National Institutes of Health." Prior to issuing a press release concerning the outcome of this research, please notify the NIH awarding IC in advance to allow for coordination.

Award recipients must promote objectivity in research by establishing standards that provide a reasonable expectation that the design, conduct and reporting of research funded under NIH awards will be free from bias resulting from an Investigator's Financial Conflict of Interest (FCOI), in accordance with the 2011 revised regulation at 42 CFR Part 50 Subpart F. The Institution shall submit all FCOI reports to the NIH through the eRA Commons FCOI Module. The regulation does not apply to Phase I Small Business Innovative Research (SBIR) and Small Business Technology Transfer (STTR) awards. Consult the NIH website http://grants.nih.gov/grants/policy/coi/ for a link to the regulation and additional important information.

If you have any questions about this award, please contact the individual(s) referenced in Section IV.

Sincerely yours,

EDITH L. DAVIS
Grants Management Officer
NATIONAL INSTITUTE ON DRUG ABUSE

Additional information follows
