# Supplementary material for: Comparing a standard and tailored approach to scaling up an evidence-based intervention for antiretroviral therapy for people who inject drugs in Vietnam: study protocol for a cluster randomized hybrid type III trial
Source: Implement Sci. 2020 Aug 8;15:64. doi: 10.1186/s13012-020-01020-z (PMC7414564; doi:10.1186/s13012-020-01020-z)
Supplement: Supplementary file 2 — Additional file 2. Measurement scales. [file 13012_2020_1020_MOESM2_ESM.docx]

## Additional file 2

## Measurement scales used in SNaP study

## ORGANIZATIONAL READINESS TO CHANGE

Please indicate the extent to which you agree with each statement.

| 1 | 2 | 3 | 4 | 5 |
| --- | --- | --- | --- | --- |
| Disagree | Somewhat  Disagree | Neither Agree nor Disagree | Somewhat  Agree | Agree |

| 1. People who work here feel confident that the organization can get people invested in implementing SNaP. | 1 2 3 4 5 |
| --- | --- |
| 1. People who work here are committed to implementing SNaP. | 1 2 3 4 5 |
| 1. People who work here feel confident that they can keep track of progress in implementing SNaP. | 1 2 3 4 5 |
| 1. People who work here will do whatever it takes to implement SNaP. | 1 2 3 4 5 |
| 1. People who work here feel confident that the organization can support people as they adjust to SNaP. | 1 2 3 4 5 |
| 1. People who work here want to implement SNaP. | 1 2 3 4 5 |
| 1. People who work here feel confident that they can keep the momentum going in implementing SNaP. | 1 2 3 4 5 |
| 1. People who work here feel confident that they can handle the challenges that might arise in implementing SNaP. | 1 2 3 4 5 |
| 1. People who work here are determined to implement SNaP. | 1 2 3 4 5 |
| 1. People who work here feel confident that they can coordinate tasks so that implementation goes smoothly. | 1 2 3 4 5 |
| 1. People who work here are motivated to implement SNaP. | 1 2 3 4 5 |
| 1. People who work here feel confident that they can manage the politics of implementing SNaP. | 1 2 3 4 5 |

**IMPLEMENTATION LEADERSHIP SCALE**

**Staff Version**

Please indicate the extent to which you agree with each statement.

**Note:** Please note that the different level of agreement (from 0 to 4 instead of from 1 to 5) will be used for the following statements.

| 0 | 1 | 2 | 3 | | 4 |  |  |  |
| --- | --- | --- | --- | --- | --- | --- | --- | --- |
| Not at all | Slight extent | Moderate extent | Great extent | | Very great extent |  |  |  |
|  | **Proactive** | | | | |  | | |
| 1 | [My supervisor] has developed a plan to facilitate implementation of SNaP | | | | | 0 1 2 3 4 | | |
| 2 | [My supervisor] has removed obstacles to the implementation of SNaP | | | | | 0 1 2 3 4 | | |
| 3 | [My supervisor] has established clear department standards for the implementation of SNaP | | | | | 0 1 2 3 4 | | |
|  | **Knowledgeable** | | | | |  | | |
| 4 | [My supervisor] is knowledgeable about SNaP | | | | | 0 1 2 3 4 | | |
| 5 | [My supervisor] is able to answer my questions about SNaP | | | | | 0 1 2 3 4 | | |
| 6 | [My supervisor] knows what he or she is talking about when it comes to SNaP | | | | | 0 1 2 3 4 | | |
|  | **Supportive** | | | | |  | | |
| 7 | [My supervisor] recognizes and appreciates employee efforts toward successful implementation of SNaP | | | | | 0 1 2 3 4 | | |
| 8 | [My supervisor] supports employee efforts to learn more about SNaP | | | | | 0 1 2 3 4 | | |
| 9 | [My supervisor] supports employee efforts to use SNaP | | | | | 0 1 2 3 4 | | |
|  | **Perseverant** | | | | |  | | |
| 10 | [My supervisor] perseveres through the ups and downs of implementing SNaP | | | | | 0 1 2 3 4 | | |
| 11 | [My supervisor] carries on through the challenges of implementing SNaP | | | | | 0 1 2 3 4 | | |
| 12 | [My supervisor] reacts to critical issues regarding the implementation of SNaP by openly and effectively addressing the problem(s) | | | | | 0 1 2 3 4 | | |

**Supervisor Version**

Please indicate the extent to which you agree with each statement.

**Note:** Please note that the different level of agreement (from 0 to 4 instead of from 1 to 5) will be used for the following statements.

| 0 | 1 | 2 | 3 | | 4 |  |  |  |
| --- | --- | --- | --- | --- | --- | --- | --- | --- |
| Not at all | Slight extent | Moderate extent | Great extent | | Very great extent |  |  |  |
|  | **Proactive** | | | | |  | | |
| 1 | I have developed a plan to facilitate implementation of SNaP | | | | | 0 1 2 3 4 | | |
| 2 | I have removed obstacles to the implementation of SNaP | | | | | 0 1 2 3 4 | | |
| 3 | I have established clear department standards for the implementation of SNaP | | | | | 0 1 2 3 4 | | |
|  | **Knowledgeable** | | | | |  | | |
| 4 | I am knowledgeable about SNaP | | | | | 0 1 2 3 4 | | |
| 5 | I am able to answer my questions about SNaP | | | | | 0 1 2 3 4 | | |
| 6 | I know what I am talking about when it comes to SNaP | | | | | 0 1 2 3 4 | | |
|  | **Supportive** | | | | |  | | |
| 7 | I recognize and appreciates employee efforts toward successful implementation of SNaP | | | | | 0 1 2 3 4 | | |
| 8 | I support employee efforts to learn more about SNaP | | | | | 0 1 2 3 4 | | |
| 9 | I support employee efforts to use SNaP | | | | | 0 1 2 3 4 | | |
|  | **Perseverant** | | | | |  | | |
| 10 | I persevere through the ups and downs of implementing SNaP | | | | | 0 1 2 3 4 | | |
| 11 | I carry on through the challenges of implementing SNaP | | | | | 0 1 2 3 4 | | |
| 12 | I react to critical issues regarding the implementation of SNaP by openly and effectively addressing the problem(s) | | | | | 0 1 2 3 4 | | |

**IMPLEMENTATION CLIMATE SCALE**

Please indicate the extent to which you agree with each statement.

| 0 | 1 | 2 | 3 | 4 |
| --- | --- | --- | --- | --- |
| Not at all | Slight extent | Moderate extent | Great extent | Very great extent |

|  | Focus on SNaP |  |
| --- | --- | --- |
| 1 | One of this team/agency’s main goals is to use SNaP effectively | 0 1 2 3 4 |
| 2 | People in this team/agency think that the implementation of SNaP is important | 0 1 2 3 4 |
| 3 | Using SNaP is a top priority in this team/agency | 0 1 2 3 4 |
|  | Educational Support for SNaP |  |
| 4 | This team/agency provides conferences, workshops, or seminars focusing on SNaP | 0 1 2 3 4 |
| 5 | This team/agency provides SNaP trainings or in-services | 0 1 2 3 4 |
| 6 | This team/agency provides SNaP training materials, journals, etc | 0 1 2 3 4 |
|  | Recognition for SNaP |  |
| 7 | Clinicians in this team/agency who use SNaP are seen as clinical experts | 0 1 2 3 4 |
| 8 | Clinicians who use SNaP are held in high esteem in this team/agency | 0 1 2 3 4 |
| 9 | Clinicians in this team/agency who use SNaP are more likely to be promoted | 0 1 2 3 4 |
|  | Rewards for SNaP |  |
| 10 | This team/agency provides financial incentives for the use of SNaP | 0 1 2 3 4 |
| 11 | The better you are at using SNaP, the more likely you are to get a bonus or a raise | 0 1 2 3 4 |
| 12 | This team/agency provides the ability to accumulate compensated time for the use of SNaP | 0 1 2 3 4 |
|  | Selection for SNaP |  |
| 13 | This team/agency selects staff who have previously used SNaP | 0 1 2 3 4 |
| 14 | This team/agency selects staff who have had formal education supporting SNaP | 0 1 2 3 4 |
| 15 | This team/agency selects staff who value SNaP | 0 1 2 3 4 |
|  | Selection for Openness |  |
| 16 | This team/agency selects staff who are adaptable | 0 1 2 3 4 |
| 17 | This team/agency selects staff who are flexible | 0 1 2 3 4 |
| 18 | This team/agency selects staff open to new types of interventions | 0 1 2 3 4 |
|  |  |  |

**THE OTTAWA ACCEPTABILITY OF DECISION RULES INSTRUMENT**

**(for site staff participants)**

Please indicate the extent to which you agree with each statement.

**Note:** Please note that the different level of agreement (from 1 to 7 instead of from 0 to 4) will be used for the following statements.

| **Please indicate your level of agreement with each of the following statements about SNaP.** | **1**  **Strongly disagree** | **2**  **Moderately disagree** | **3**  **Slightly disagree** | **4**  **Slightly agree** | **5**  **Moderately agree** | **6**  **Strongly agree** | **7**  **No opinion/Don’t know** |
| --- | --- | --- | --- | --- | --- | --- | --- |
| 1. SNaP is easy to use | _(1)_ | _(2)_ | _(3)_ | _(4)_ | _(5)_ | _(6)_ | _(7)_ |
| 1. SNaP is easy to remember | _(1)_ | _(2)_ | _(3)_ | _(4)_ | _(5)_ | _(6)_ | _(7)_ |
| 1. SNaP is useful in my practice | _(1)_ | _(2)_ | _(3)_ | _(4)_ | _(5)_ | _(6)_ | _(7)_ |
| 1. The wording of SNaP is clear and unambiguous. | _(1)_ | _(2)_ | _(3)_ | _(4)_ | _(5)_ | _(6)_ | _(7)_ |
| 1. My colleagues support use of SNaP | _(1)_ | _(2)_ | _(3)_ | _(4)_ | _(5)_ | _(6)_ | _(7)_ |
| 1. Patients benefit from use of SNaP. | _(1)_ | _(2)_ | _(3)_ | _(4)_ | _(5)_ | _(6)_ | _(7)_ |
| 1. I’m already using another similar strategy. | _(1)_ | _(2)_ | _(3)_ | _(4)_ | _(5)_ | _(6)_ | _(7)_ |
| 1. The environment I work in makes it difficult to use SNaP. | _(1)_ | _(2)_ | _(3)_ | _(4)_ | _(5)_ | _(6)_ | _(7)_ |

# **ACCEPTABILITY TO INTERVENTION MEASURE**

# **(for people who inject drugs participants)**

| **[INTERVIEWER: READ]** *I am going to ask you about your opinions about the SNaP intervention. For the following questions I will ask you to tell me how much you agree or disagree with the following statements. Use this response scale ranging from 1 (“completely disagree”) to 5 (‘‘completely agree”).* | | | | | |
| --- | --- | --- | --- | --- | --- |
|  | Completely disagree | Disagree | Neither agree nor disagree | Agree | Completely agree |
| 1. The SNaP intervention meets my approval. | ➀ | ➁ | ➂ | ➃ | ➄ |
| 2. The SNaP intervention is appealing to me. | ➀ | ➁ | ➂ | ➃ | ➄ |
| 3. I like the SNaP intervention. | ➀ | ➁ | ➂ | ➃ | ➄ |
| 4. I welcome the SNaP intervention. | ➀ | ➁ | ➂ | ➃ | ➄ |
